# Diagnostic difficulties of AH1N1 influenza infection in children with acute lymphoblastic leukemia: Two case reports

**DOI:** 10.1097/MD.0000000000022790

**Published:** 2020-10-23

**Authors:** Joanna Zawitkowska, Monika Lejman, Katarzyna Drabko, Agnieszka Zaucha-Prażmo

**Affiliations:** a Department of Paediatric Haematology, Oncology, and Transplantology, Medical University; b Laboratory of Genetic Diagnostics, Medical University of Lublin, Poland.

**Keywords:** chemotherapy, childhood acute lymphoblastic leukemia, the swine-origin influenza A influenza

## Abstract

**Introduction:**

Children with acute lymphoblastic leukemia (ALL) are at a risk of developing influenza-related complications. Approximately 10% of influenza-infected children with ALL or other types of cancer need intensive care, and 5% of them eventually die.

**Patients’ concerns:**

We report 2 children with ALL and the swine-origin influenza A virus infection. Diagnosing influenza in them was a challenge. Medical records of these children were reviewed for demographic, clinical, and laboratory data. Patients were hospitalized in the Department of Pediatric Hematology, Oncology, and Transplantology of the Medical University of Lublin, Poland. Case 1 involved a 2-year-old girl who, according to acute lymphoblastic leukemia intercontinental Berlin-Frankfürt-Münster protocol 2009, started chemotherapy in July 2015. She was categorized in the intermediate risk group and received the induction and consolidation phase of the therapy without severe complications. The reinduction therapy was administered in the outpatient department till the 15^th^ day. On the 20^th^ day of this phase, she was admitted to our department with fever, mucositis, tachypnea, abdominal pain, and diarrhea. In September 2009, a 14-year-old boy (case 2) who, according to acute lymphoblastic leukemia intercontinental Berlin-Frankfürt-Münster protocol 2002, was categorized in the high-risk (HR) group, received the induction (Protocol I) phase of therapy without severe complications. On the 7^th^ day of the HR-1 course, he manifested fever and strong, tiring cough, followed by strong mucositis. Chemotherapy had to be interrupted in both children.

**Diagnosis:**

Respiratory viral infections, causing pneumonia, occurred in both patients during anticancer treatment. Initially, the real-time polymerase chain reaction test for the swine-origin influenza A was negative in both patients, which delayed the diagnosis. Additionally, bacterial, and fungal complications were also observed.

**Interventions:**

Both patients received oseltamivir twice a day, a broad-spectrum antibiotic, antifungal drug, and granulocyte colony growth factor.

**Outcomes:**

The disease progressed quickly, and our patients subsequently died.

**Conclusion:**

We speculated that early antiviral treatment can help in the better management of patients in the HR group. It is also important to minimize influenza morbidity and mortality by vaccinating family members, using empiric therapy, providing immediate antiviral therapy, and educating parents about hygiene measures.

## 1 Introduction

Influenza is one of the most widespread and significant viral infection that infects 10% to 20% of the population, of all age groups every year. A global pandemic caused by the swine influenza A (H1N1) virus was reported in 2009. The virus infects the upper and lower respiratory tract causing symptoms mediated by the production of interferons and cytokines. Real-time polymerase chain reaction (RT-PCR) has the highest sensitivity and specificity for the test. Diagnostic difficulties may occur in immunocompromised patients and during multipathogenic infections.^[1]^

Children with acute lymphoblastic leukemia (ALL) are particularly at a risk of influenza-associated complications. Respiratory viral infections, causing respiratory failure, pneumonia, and need for ventilatory support, occur in 10% to 20% of the patients during cancer therapy.^[1,2]^ Additional complications of influenza include: bacteremia, affecting 16.5% of the children, and less common fungal pneumonias or hemophagocytic lymphohistiocytosis (HLH).^[2,4,5]^ Antiviral treatment is recommended (preferably within 48 hour) for patients with confirmed or suspected flu having serious infection or an increased risk of complications. Influenza in children with cancer causes a significant delay in chemotherapy in 20% to 80% of the patients (average treatment interruptions of ≥3 week), which in turn affects the prognosis.^[1]^ Approximately 10% of influenza-infected children with ALL or other types of cancer need intensive care, and death occurs in eventually 5% of the patients.^[1,3]^

We report 2 patients with ALL and the swine-origin influenza A (AH1N1 virus infection and a challenging diagnosis of influenza. Medical records of these children were reviewed for demographic, clinical, and laboratory data. Patients were hospitalized in the Department of Pediatric Hematology, Oncology, and Transplantology of the Medical University of Lublin, Poland.

## 2 Case reports

### 2.1 Case 1

A 2-year-old girl was admitted to our hospital because of petechiae and bruising on the lower extremities and splenomegaly. There were no comorbidities, such as obesity, diabetes, bronchial asthma, and no significant family history. Written informed consent was obtained from the patient's parents for publication of this case report and accompanying images.

**Figure 1:**
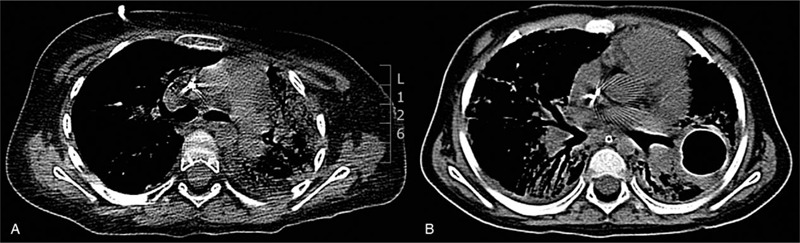
Computer tomography scan of the chest of the first child when clinical symptoms appeared (A) and after another 2 months (B).

The child was diagnosed with B-cell precursor common positive ALL and according to acute lymphoblastic leukemia intercontinental Berlin-Frankfürt-Münster protocol (ALL IC-BFM 2009, chemotherapy was started in July 2015.^[6]^ She was categorized in the intermediate risk group (white blood cell count (WBC) was <20 000/μL; she showed a good response to steroids, as on day 8 the blast count in peripheral blood was <1000/μL; myelogram on day 15 had 2.4% blasts and minimal residual disease was 1.94%; myelogram on day 33 had 4.8% blasts). She received the induction (Protocol I) and consolidation (Protocol M) phase of therapy without any severe complications. The reinduction therapy was administered in the outpatient department till day 15. Chemotherapy was well tolerated. On day 20 of this phase, she was admitted to our department due to fever, mucositis, tachypnea, abdominal pain, and diarrhea. Laboratory and microbiological data are presented in a time-dependent manner in Table 1. Chemotherapy was interrupted, and computed tomography (CT) of the chest showed massive inflammatory parenchymal lesions mainly in the left lung with the picture of “frosted glass” and mediastinal shift to the left (Fig. 1A). On admission, the nasopharyngeal swab for influenza A, AH1N1, and B using RT-PCR method was negative. Due to the likelihood of mixed bacterial-fungal etiology, we decided to administer broad-spectrum antibiotics (meropenem and vancomycin) and antifungal drug (liposomal amphotericin B). Due to neutropenia, granulocyte colony growth factor was used in the supportive care. Unfortunately, after 6 days, she developed respiratory failure and was transferred to the intensive care unit (ICU). The antibiotic and antifungal therapy were continued but did not prove beneficial. Peripheral blood and bronchoalveolar lavage (BAL) were analyzed for respiratory pathogens and *Aspergillus fumigatus* deoxyribonucleic acid by using the PCR method. Infection with the AH1N1 virus was confirmed only in the BAL fluid, and 45 mg oseltamivir was administered daily. This was continued for 2 weeks without any effect. Due to the progression of clinical symptoms, respiratory pathogens, and *A fumigatus* deoxyribonucleic acid tests were repeated, and the AH1N1 test was positive in both the blood and BAL that time. CT showed that manifestations persisted with additional pulmonary cavities. Therefore, amantadine sulfate was administered with oseltamivir. After 4 weeks of therapy, CT presented massive progression in the changes and enlargement of the pulmonary cavities (Fig. 1B). The patient remained in the ICU for 3 months and eventually succumbed to pulmonary hemorrhages.

**Table 1:**
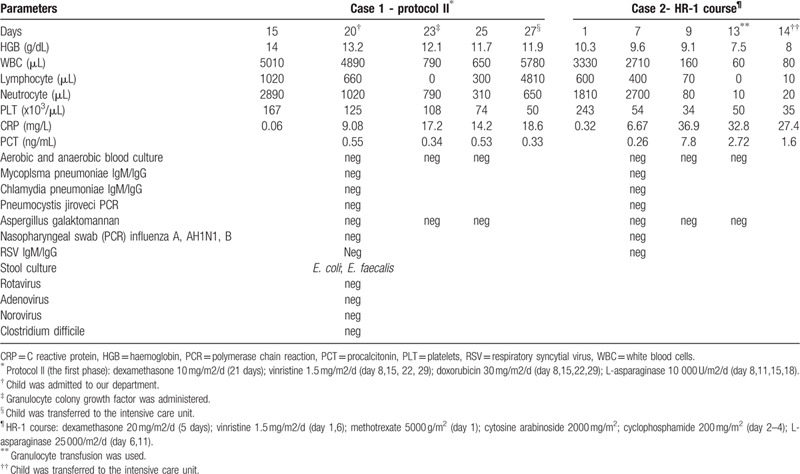
Laboratory and microbiological data in a time-dependent manner in the first and second case.

### 2.2 Case 2

A 14-year-old boy was admitted to our hospital because of petechiae and bruising on the lower extremities, hepatosplenomegaly, and facial left nerve palsy. Comorbidity included obesity but no diabetes, bronchial asthma, or any significant family history were noted. Written informed consent was obtained from the patient's parents for publication of this case report and accompanying images.

**Figure 2:**
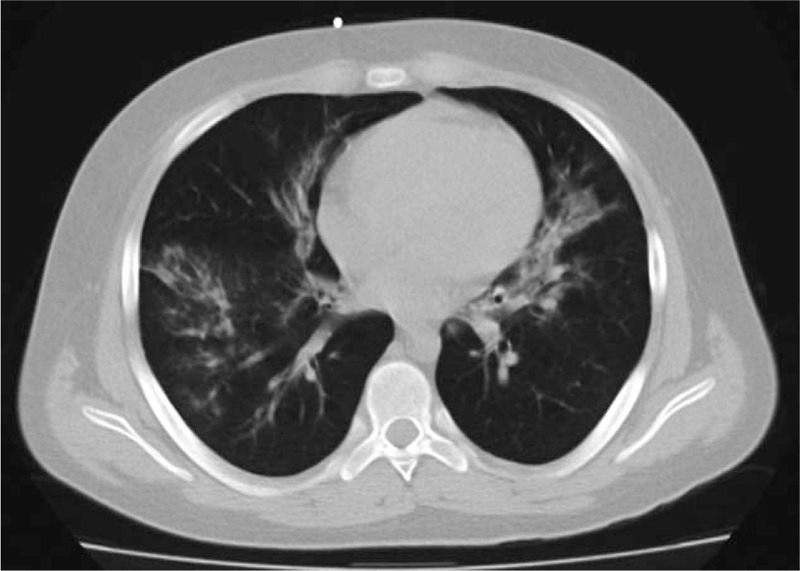
Computed tomography of the chest of the second child when clinical symptoms appeared.

The child was diagnosed with B-cell precursor common positive ALL with infiltration of the central nervous system (pleocytosis of 145/μL), and according to ALL IC-BFM 2002, treatment was started in September 2009.^[7]^ He was categorized in the high-risk (HR group (WBC count on diagnosis was >20 000/μL; he showed poor response to steroids as on day 8 the blast count in peripheral blood was >1000/μL; myelogram on day 15 had 14% blasts and myelogram on day 33 had 4.8% blasts). He received the induction (Protocol I) phase of therapy without severe complications. On the 7^th^ day of the HR-1 course, fever, and a strong, tiring cough occurred, followed by strong mucositis. Chemotherapy had to be interrupted. Laboratory and microbiological data are presented in a time-dependent manner in Table 1. Initially, the RT-PCR test for AH1N1 was negative. CT of the chest showed massive banded and consolidated densities on both sides (Fig. 2). The changes may correspond to bacterial and fungal infection. Broad-spectrum antibiotics (cilastatin, imipenem, and vancomycin), an antifungal drug (liposomal amphotericin B) and granulocyte colony growth factor were administered. Additionally, intravenous immunoglobulin was also administered. Due to lack in hematological recovery and constantly deteriorating condition of the child, granulocytes were transfused. This procedure was started after 7 days from the appearance of clinical symptoms. On the day following transfusion, respiratory failure occurred, and the child was transferred to the ICU. BAL analysis was performed and infection with the AH1N1 virus was diagnosed. The patient received oseltamivir 75 mg twice a day. Despite the transfusion, the child died due to multiple organ failure after 10 days of the antiviral therapy. [insert Fig. 2].

## 3 Discussion

The influenza virus, particularly AH1N1, is well known to become lethal in ALL children undergoing chemotherapy. This virus spreads easily in pediatric oncology departments from both visitors and health care workers.^[1,8]^

Yöntem Y et al reported that 8 out of the 98 children who received chemotherapy for leukemia were diagnosed with the pandemic H1N1 infection. In our study, 2 out of 8 patients had a fatal course (25%) of which 1 developed pneumonia and acute respiratory distress syndrome, and the other developed HLH and died due to secondary infection in the 6^th^ week of treatment for HLH.^[9]^

In another study, authors reported 10 patients with a median age of 7 years who suffered from ALL and were treated for influenza in an institution. They were all treated with oseltamivir. H1N1 RT-PCR of the nasopharyngeal aspirate was carried out whenever influenza was suspected. Oseltamivir was generally given empirically without awaiting microbiological confirmation, except in the first patient in whom the treatment began after 5 days following fever. No deaths were reported. Two patients under intensive chemotherapy developed pneumonia and 1 required ventilatory support. ALL patients under maintenance treatment developed a mild disease.^[10]^

When the first patient had infection, influenza was not reported in the hospital, neither among patients nor the staff. It is highly probable that the child was infected at home, even though the parents did not have information that someone was sick in their surroundings. The girl was undergoing intensive steroid treatment and chemotherapy, which were significant risk factors. Despite bacterial, viral, and fungal tests, it was difficult to diagnose an AH1N1 viral infection. Only the BAL fluid specimen confirmed viral infection. Antiviral treatment was applied after 48 hours from the appearance of first clinical symptoms. The additional complications of influenza comprised bacterial and fungal infections The presence of the virus in the blood in the next test confirmed the ineffectiveness of antiviral treatment.

In 2009, when the second patient developed an infection, WHO (World Health Organization) declared H1N1 influenza as a pandemic. The patient was the first to have flu diagnosed in our clinic at that time. Next, 5 out of the 25 cancer children and 1 doctor contracted pneumonia caused by AH1N1. Our patient received an aggressive course of chemotherapy, and therefore progression of infection was very quick. Like the previous child, only BAL tested positive. Oseltamivir was administered after 9 days from the initiation of clinical symptoms. Immediately, the remaining children and the doctor with clinical symptoms were given empiric antiviral therapy (not awaiting microbiological confirmation). All patients responded well to the treatment. In these individuals, the nasopharyngeal aspirates confirmed (PCR) the AH1N1 infection.

In the analyzed patients, the tests were negative, which delayed the diagnosis. The reason could be severe mucositis in both children. In our opinion, patients with mucositis, serious clinical symptoms, and negative nasopharyngeal swab test results should undergo BAL analysis. We speculate that early empirical antiviral treatment could produce better results in HR groups.

Most studies show a link between immunosuppression and the development of influenza-associated pneumonia. Unfortunately, because of immunosuppression, vaccines, such as the seasonal influenza vaccine, hold very little benefit for immunosuppressed children. Therefore, it is important to minimize influenza morbidity and mortality by using empiric therapy and early initiation of antiviral therapy and educating parents about hygiene measures. The education and vaccination of families and medical staff dealing with immunocompromised patients is of utmost importance.^[1]^

## Author contributions

JZ, ML, AZP, KD were responsible for the study design. ML, AZP, KD collected the clinical data.

JZ analysed the data interpreted the data, wrote, and supervised the paper. All authors read and approved the final manuscript.
